# Anti-ganglioside Antibodies in Guillain-Barre Syndrome: A Novel Immunoblotting-Panel Assay

**DOI:** 10.3389/fneur.2021.760889

**Published:** 2021-11-25

**Authors:** Jiting Zhu, Yuanyuan Zhang, Runyun Li, Yi Lin, Ying Fu, Yaping Yan, Wenli Zhu, Ning Wang, Zaiqiang Zhang, Guorong Xu

**Affiliations:** ^1^Department of Neurology and Institute of Neurology, The First Affiliated Hospital of Fujian Medical University, Fuzhou, China; ^2^National Engineering Laboratory for Resource Development of Endangered Crude Drugs in Northwest of China, College of Life Sciences, Shaanxi Normal University, Xi'an, China; ^3^Department of Neurology, China National Clinical Research Center for Neurological Diseases, Beijing Tiantan Hospital, Capital Medical University, Beijing, China

**Keywords:** Guillain-Barre syndrome, immunoblotting detection, ganglioside, antibodies, peripheral neuropathy

## Abstract

**Objective:** This study aimed to determine the diagnostic efficiency of a novel immunoblotting detection assay for anti-ganglioside antibodies (AGAs) in the Guillain–Barre syndrome (GBS).

**Method:** Serum immunoglobulin (IgG and IgM) of AGAs were measured in 121 participants from a registered cohort study of immune-mediated neuropathies and 29 healthy controls by immunoblotting panel assay. Sensitivity, specificity, and positive predictive value (PPV) of the assay were compared to calculate the diagnostic accuracy.

**Result:** In our cohort, any of the AGAs were positive in 42.4% of the GBS patients. The sensitivity and specificity of AGAs (both IgG and IgM) in the diagnosis of GSB were 42 and 76% while for IgG-AGAs were 35 and 87%. AGAs positivity had a significant association with the AMAN subtype (*P* = 0.0004), and the sensitivity, specificity of AGAs in AMAN were 86, 69%, respectively with high (AUC = 0.78, *p* = 0.002) discriminative powers. GM1-IgG AGA was more common and specific to AMAN patients than other GBS forms (*p* = 0.008).

**Conclusion:** Our novel immunoblotting detection assay could complement GBS diagnosis. IgG-AGAs were more likely to be detected in GBS, and GM1-IgG AGA could assist AMAN diagnosis.

## Introduction

Guillain-Barre syndrome (GBS) is a potentially life-threatening immune-mediated peripheral neuropathy usually triggered by infections and with an approximate global incidence of 1–2/100,000 person-years (1). GBS is characterized by acute and progressive weakness and sensory loss, usually followed by slow clinical recovery. Approximately 20% of the patients with GBS develop respiratory failure and require mechanical ventilation, while 3–10% succumb to disease. In addition, many patients experience long-lasting disability and complaints even with the best medical care (2). This undesirable outcome is partly due to the lack of an effective method for timely diagnosis. Current diagnostic strategies of GBS are based on the clinical symptoms, electrophysiological and cerebrospinal fluid (CSF) examinations (3–5), without any specific diagnostic biomarkers.

Guillain-Barre syndrome is thought to be caused by an aberrant immune response to infections that results in damage to the peripheral nerves, but a clear pathogenesis mechanism remains elusive. Interestingly, the clinical and serological data clearly shows a disease-specific correlation between peripheral neuropathies and particular anti-glycolipid antibodies (6, 7). Infection with *Campylobacter jejuni* can elicit bacterial lipo-oligosaccharides antibodies cross-reactive with axon gangliosides, a process known as molecular mimicry. Furthermore, anti-gangliosides antibodies (AGAs) have been reported in the patients with GBS, particularly in acute motor axonal neuropathy (AMAN), acute motor and sensory axonal neuropathy (AMSAN), MFS. Currently, two methodological approaches are available for AGAs detection; ELISA (either inhouse or commercial) and line-/dot-blot (commercial) (8). Testing AGAs panels by immunoblotting is simpler and more cost effective than ELISA, testing for the reactivity against different peripheral nerve antigens in a single assay. This study measured serum AGAs with a new immunoblotting panel in a prospective immune-mediated peripheral neuropathy cohort, aiming to evaluate its applicability in the clinical diagnosis.

## Materials and Methods

### Patients and Study Design

Patients were recruited from a cohort study of immune-mediated peripheral neuropathy (A Registered Cohort Study of Immune-Mediated Neuropathies, RCSIMN Cohort) (ClinicalTrials.gov NCT:04292834) at the Neurogenetic Diseases Center in First Affiliated Hospital, the Fujian Medical University and Beijing Tiantan Hospital, Capital Medical University between Jan 2017 and Jan 2020. The study protocol and informed consent procedures were approved by the institutional review boards and ethics committee at First Affiliated Hospital of Fujian Medical University. Written informed consent was provided by all the participants (121 patients with immune-mediated neuropathies and 29 healthy controls).

All patients met the diagnostic criteria of various inflammatory peripheral neuropathy (5, 9, 10), and were included in the study within 2 weeks of symptoms onset. Patients with severe complications, severe mental disorders, or under 18 years old were excluded. The follow-up period was at least 6 months for all the patients.

### Sample Collection and Immunodot Assays

Samples of standardized patients were collected in the EDTA-containing tubes and processed for 10 min centrifugation at 1,500 g, 20°C. Sera was stored at −80°C and documented in the specimen registry of our institute. Plasma samples were anonymized, randomized, and measurements were carried out by a blinded testier.

We employed a ganglioside autoantibody detection kit (CAT. No.: MT164-16). Purified gangliosides were bound in a polyvinylidene difluoride (PVDF) membrane. Individual gangliosides dissolved with the organic solvents. The secondary antibody, alkaline phosphatase (AP)-conjugated anti-human IgG and IgM was incubated for recognizing human serum antibodies, then the chromogenic reaction was carried out by the enzyme reaction substrate to form visible speckled chromogenic reaction. Twelve kinds of antibodies could be detected simultaneously, including GM1, GM2, GM3, GM4, GQ1b, GT1b, GT1a, GD1a, GD1b, GD2, GD3, and sulfatide. A positive autoantibody against ganglioside is judged based on whether the coating region has a speckled coloring. The positive result was indicated: the antigen-coated area presents a clearly distinguishable circular or approximately circular, similar to the light blue-gray or dark blue-black, with a darker color than the blank control. The negative result was indicated: the color of the antigen-coated area is shallower or equivalent to the blank control area, or that of the antigen-coated area is slightly darker than the blank control. Any AGA positive out of 12 items was defined as positive for this test. Two investigators read the results without knowing one another's work. We repeated 65 samples randomly to verify the results of our experiment.

### Statistical Analysis

Categorical variables were as described by the counts and percentages, and were compared by chi-squared test and Fisher exact test. Multiple regression analysis was used to control for potentially confounding factors (gender, age, premorbid inducement, anamnesis, symptom, severity, and subtype) that may influence the positive incidence of AGAs. Receiver operating characteristic (ROC) curve analyses were performed to assess diagnostic sensitivity and specificity of AGAs detection. Statistical significance was defined as *p* < 0.05. Statistical analysis was performed using an SPSS version 26.0 software (SPSS, Inc.).

## Results

### Cohort Study

**Figure 1:**
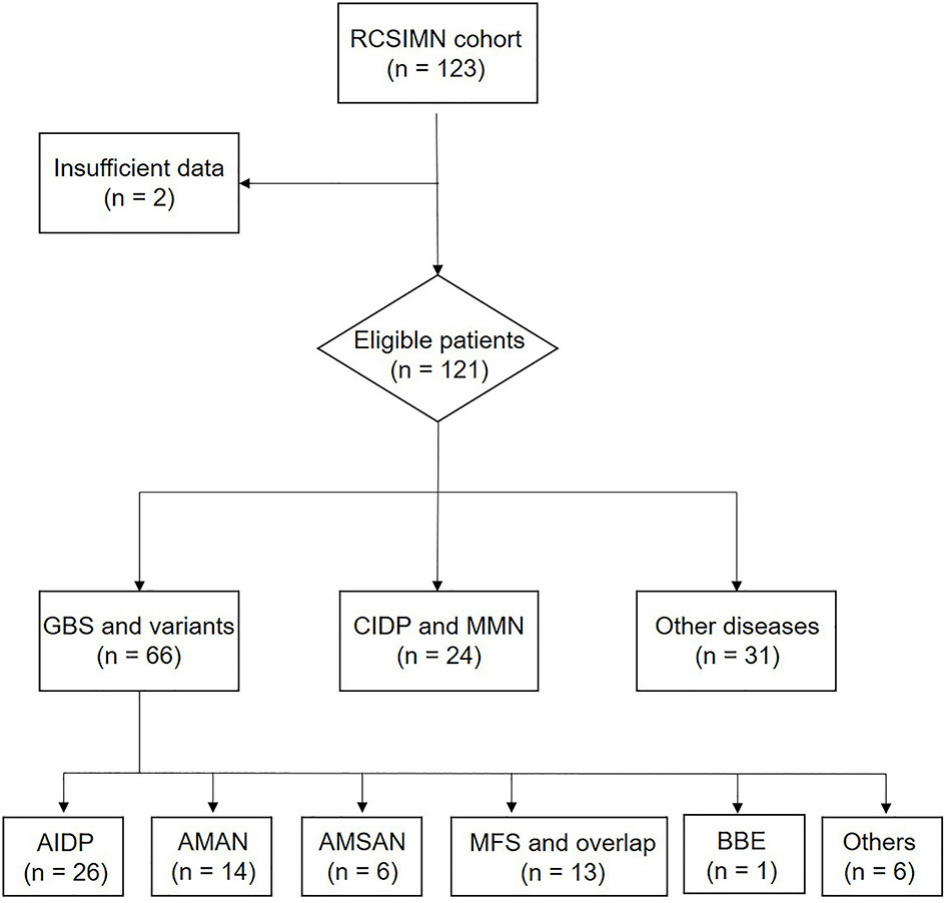
Trial profile. RCSIMN, A Registered Cohort Study of Immune-Mediated Neuropathies; GBS, Guillain-Barre syndrome; CIDP, Chronic-inflammatory demyelinating polyneuropathy; AIDP, Acute inflammatory demyelinating polyradiculoneuropathy; AMAN, Acute motor axonal neuropathy; AMSAN, Acute motor sensory axonal neuropathy; MFS, Miller-Fisher syndrome; BBE, Bickerstaff brainstem encephalitis.

Registered Cohort Study of Immune-Mediated Neuropathies Cohort data are displayed in Figure 1. Two cases were excluded because of the incompetent information, leading to a total of 121 serum samples for testing. A total of 66 patients with GBS were presented as: acute inflammatory demyelinating polyneuropathies (AIDP, *N* = 26), acute motor axonal neuropathy (AMAN, *N* = 14), acute motor-sensory axonal neuropathy (AMSAN, *N* = 6), Miller-Fisher syndrome and overlap (MFS, *N* =13), Bickerstaff brainstem encephalitis (BBE, *N* = 1), or other variants (*N* = 6). These were compared with 22 patients with a chronic inflammatory demyelinating polyneuropathy and two patients with multifocal motor neuropathy (MMN, *N* = 2), and a number of other diseases mimicking the immune-mediated neuropathy diseases (OND) (*N* = 31). All the patients presented similar symptomatology with early atypia with the forms of limb weakness or numbness, including paraneoplastic syndrome, motor neuron disease, vascular inflammatory peripheral neuropathy, and so on.

### Detection-Kit Evaluating

**Figure 2:**
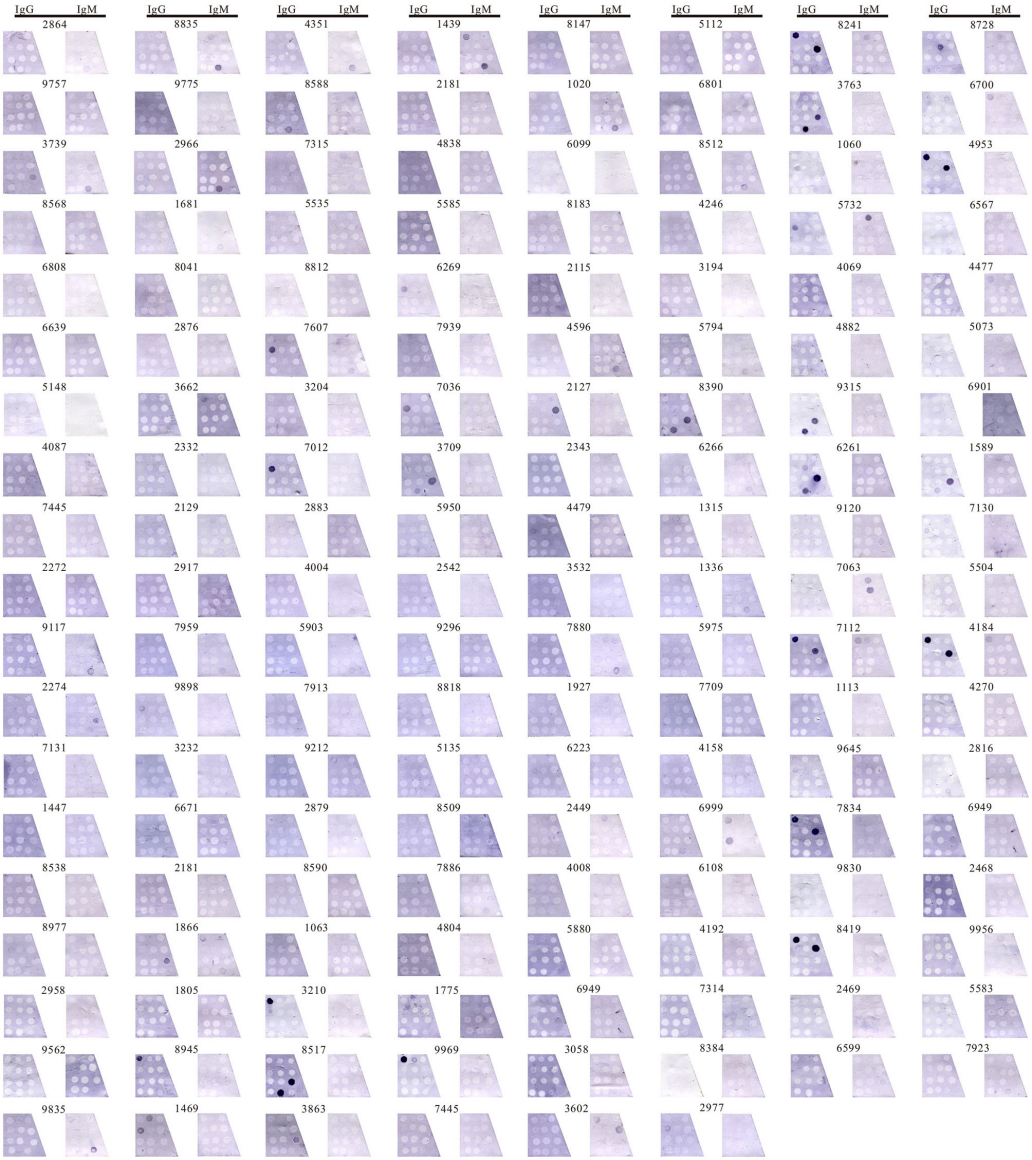
Detecting anti-ganglioside antibodies in 150 participants. Detection IgG-AGAs and IgM-AGAs in 150 samples by immunoblotting assay, including GM1, GM2, GM3, GM4, GD1a, GD1b, GD2, GD3, GQ1b, GT1b, GT1a, and sulfatide.

Two investigators read the results without knowing it in advance. The interrater agreement between investigator 1 and investigator 2 was 0.759 (*p* < 0.001) translating to an almost perfect agreement as measured by Cohen's Kappa. These results were validated with a subset of 65 samples that were selected randomly for recount leading to an interrater agreement value of 0.753 (*p* < 0.001) between experiment 1 and experiment 2. The entire results were showed in Figure 2.

### Frequency of Anti-ganglioside Antibody in Patients With GBS

Antiganglioside antibodies including IgM and IgG were found in the patients with GBS (*N* = 7; 10.6%, *N* = 23; 34.8%), while any of the AGAs were positive in 42.4% of the GBS group. The sensitivity and specificity of IgG-AGA assay in the diagnosis of GSB were 35% (95% CI 24–48) and 87% (95% CI 77–93), with a positive (PPV) and negative predictive values (NPV) of 68 and 63%, respectively. The positive likelihood ratio was 2.66 (95% CI 1.40–5.06), and the AUC reported for GBS vs. disease controls for IgG-AGAs was 0.61, *p* = 0.040 (Table 1).

**Table 1:** Antibody testing results.

	**Sensitivity (CI 95%)**	**Specificity (CI 95%)**	**Positive predictive value (%)**	**Negative predictive value (%)**	**Positive likelihood ratios (CI 95%)**	**AUC**
Antibody testing in total patients with GBS						
IgG-AGAs	35 (24, 48)	87 (77, 93)	68	63	2.66 (1.40, 5.06)	0.61, *P* = 0.040
IgG- and IgM-AGAs	42 (31, 55)	76 (65, 85)	58	63	1.78 (1.11, 2.86)	0.59 *P* = 0.047
Antibody testing in patients with AMAN						
IgG-AGAs	64 (36, 86)	73 (59, 84)	39	88	2.39 (1.32, 4.33)	0.69, *P* = 0.033
IgG- and IgM-AGAs	86 (56, 97)	69 (55, 81)	43	95	2.79 (1.76, 4.41)	0.78, *P* = 0.002

*AUC, area under the curve IgG-AGAs*.

Together, IgG- and IgM-AGAs assays showed a sensitivity and specificity of 42% (95% CI 31–55) and 76% (95% CI 65–85) in the diagnosis of GBS, with PPV) and NPV of 58 and 63%, respectively. The positive likelihood ratio was 1.78 (95% CI 1.11–2.86) and AUC reported for GBS vs. disease controls was 0.59, *p* = 0.047 (Table 1).

We also detected AGAs in one of 24 patients with CIDP and MMN (4.2%), in six of 31 with OND (19.4%), and in four of 29 HCs (13.8%). IgM-AGA was more frequent in the chronic demyelinating peripheral neuropathy such as MMN and CIDP, while in amyotrophic lateral sclerosis (ALS) and other diseases, IgG-AGA was the main type. In the healthy population, the detection frequency of IgG-AGA was significantly lower than that of IgM-AGA. Of the 12 tested analytes, IgG antibodies against GM1, GT1b, GQ1b, and GD1b subtypes have a higher distribution rates, and GM1, GD1b-IgG, GT1a, and GQ1b-IgG complex antibodies had the highest detection rates in GBS, but were not related to disease severity.

### Frequency of Anti-ganglioside Antibody in Patients With AMAN

**Figure 3:**
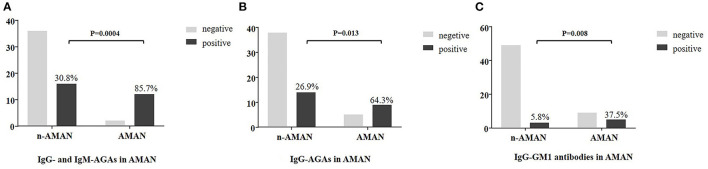
Plasma anti-ganglioside antibodies frequency in AMAN and non-AMAN patients. Frequency of anti-ganglioside antibodies is shown in the histogram. Comparison analysis was performed between AMAN and non-AMAN patients with chi-squared test. **(A)** AGAs (IgG or IgM antibodies against GM1, GM2, GM3, GM4, GQ1b, GT1b, GT1a, GD1a, GD1b, GD2, GD3, and sulfatide); **(B)** IgG-AGA (IgG antibodies against GM1, GM2, GM3, GM4, GQ1b, GT1b, GT1a, GD1a, GD1b, GD2, GD3, and sulfatide); **(C)** IgG-GM1 (IgG antibodies against GM1).

Multiple regression analysis showed that AMAN was significant correlated with serum AGAs (*p* = 0.005), while no such association was found with gender, age, premorbid inducement, anamnesis, motor involvement, superficial sensibility involvement, deep sensation involvement, or GBS disability scores (a scale used to evaluate the severity of disease) (Table 2). IgG- and IgM-AGAs, IgG-AGA, IgG-GM1 antibodies were more common in the patients with AMAN, and was specific for the diagnosis of AMAN (*p* = 0.0004, *p* = 0.013, *p* = 0.008, chi-squared test) (Figure 3).

**Table 2:** Characteristics comparison between patients with AGAs or without.

	**Patients with AGA (*n* = 28)**	**Patients without AGA(*n* = 38)**	***P-*value**
Male	17 (60.7)	23 (60.5)	0.700
Age, year	49 ± 17	47 ± 17	0.320
AMAN	12 (42.8)	2 (5.3)	0.005
Premorbid inducement	18 (64.3)	26 (68.4)	0.130
Anamnesis	15 (53.6)	25 (65.8)	0.140
Motor involvement	24 (85.7)	32 (84.2)	0.500
Superficial sensibility involvement	12 (42.9)	22 (57.9)	0.760
Deep sensation involvement	6 (21.4)	7 (18.4)	0.950
GBS disability scores >2	23 (82.1)	30 (78.9)	0.420

*Data are shown as n (%) or mean ± SD*.

*AMAN, Acute motor axonal neuropathy; GBS, Guillain-Barre syndrome; AGA, anti-ganglioside antibodies*.

In the patients with AMAN, IgG-AGA detection assay showed 64% (95% CI 36–86) sensitivity and 73% (95% CI 59–84) specificity, with PPV and NPV of 39 and 88%, respectively. The positive likelihood ratio was 2.39 (95% CI 1.32~4.33) and the AUC reported for AMAN vs. other GBS forms was 0.69, *p* = 0.033 (Table 1). When considered together, IgG- and IgM-AGAs sensitivity, specificity, PPV, NPV were 86 (95% CI 56~97), 69 (95% CI 55~81), 43, 95%, respectively, with high-discriminative powers (AUC = 0.78, *p* = 0.002) (Table 1).

### Anti-gangliosides Antibody Distribution in Variants of GBS

**Figure 4:**
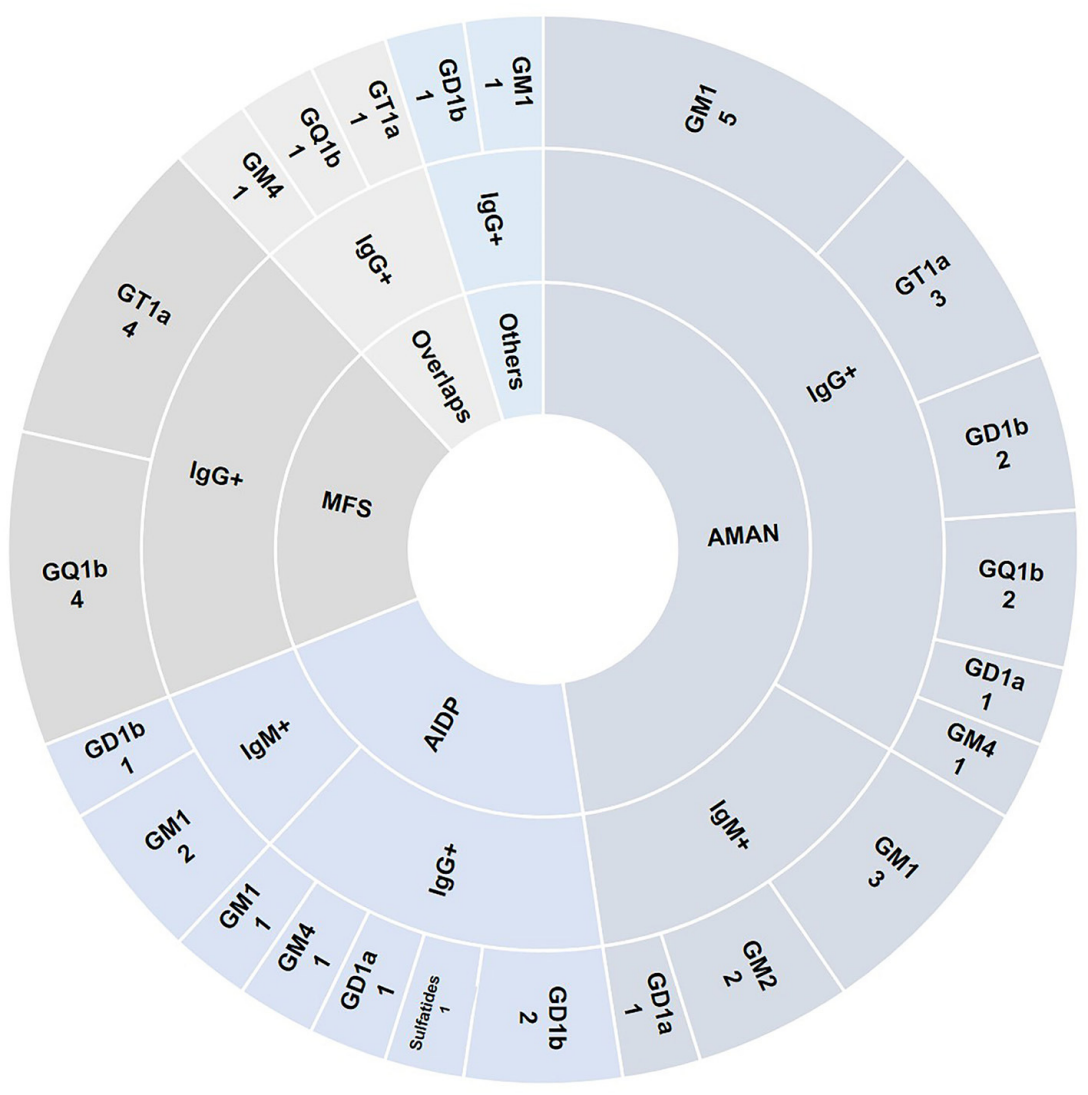
Anti-gangliosides antibody distribution in variants of GBS. Specific antibody distribution including IgG and IgM detected in 66 patients with GBS were shown in the pie chart.

Antibodies were noted against single gangliosides in the different subtypes of GBS. We detected GM1 (*N* = 8), GM2 (*N* = 2), GM4 (*N* = 1), GD1a (*N* = 2), GD1b (*N* = 2), GT1a (*N* = 3), and GQ1b (*N* = 2) in patients with AMAN, GM1 (*n* = 3), GM4 (*N* = 1), GD1a (*N* = 1), GD1b (*N* = 3), and sulfatides (*N* = 1) in patients with AIDP, GM4 (*N* =1), GT1a (*N* = 5), and GQ1b (*N* = 5) in MFS and MFS overlaps, and GM1 (*N* = 1) and GD1b (*N* = 1) in other variants (Figure 4). The maximum number of AGAs were detected in AMAN, and GM1-igG was the most common. In addition, a number of AGAs were also detected in MFS, primarily GT1a and GQ1b.

## Discussion

Here, we developed a novel immunoblotting-panel assay for the detection of serum antibodies against a broad group of ganglioside proteins (GM1, GM2, GM3, GM4, GD1a, GD1b, GD2, GD3, GQ1b, GT1b, GT1a, and sulfatide) in a cohort of inflammatory peripheral neuropathy.

Immunoblotting is a simple, rapid, and efficient assay for the detection of antibodies. Here, we multiplexed to test IgG and IgM reactivity against 12 different ganglioside analytes simultaneously. The sensitivity and specificity of IgG-AGAs in patients with GBS were 35 and 87%, showing a slightly lower sensitivity and higher specificity than previous reports. That result may form a stricter criterion in selection of the patients and more precise result interpretation. However, in this study, we did not compare results using this immunoblotting technique with another established technique such as ELISA, which is a shortcoming and needs to be further clarified in the following study.

Currently, AGAs are routinely measured by the ELISA, which detects serum antibody binding to the ganglioside-coated microwells. The frequency of antibodies detected by ELISA assay varies in different studies. The specificity and sensitivity of ELISA were reported to be 32 and 97%, respectively, while an immunoblotting assay seemed to be more efficient, with values of 56 and 100%, respectively, when the immunoblotting and ELISA approaches were compared (11). The limitation associated with ELISAs for studying the antibody response against gangliosides is the highly unspecific binding in the control wells where the sample is added without ganglioside coating (background), especially when the sample is serum. The distinct advantage of immunoblotting is that one can easily measure 12 types of antibodies simultaneously at a decreased cost. ELISA is more time consuming. With ELISA, one cannot measure all AGAs easily and simultaneously due to the use of specific kits for each individual antibody, and it is inappropriate for large-scale screening due to the increased requirement for volumes of sera and lipid reagents.

Other methods include line immunoassay, agglutination immunoassay, and glycol-array (12), and so on. The positive rate for agglutination immunoassay was 24% (13). Thin layer chromatography is considered to be the gold standard, but it is often unavailable for routine diagnostics (14). A new high throughput screening of human serum cohorts for AGAs directed to the heteromeric lipid complexes that approximate their native state in the neural membranes has established (15). However, these methods are not suitable for the routine clinical practice.

There has always been interest in the prevalence of AGAs and their associations with the specific clinical phenotypes in GBS. We observed a higher frequency of IgG-AGAs compared with IgM-AGAs. Compared with GBS, the detection of antibodies seems to have greater diagnostic significance for the specific subtype AMAN. Serum AGAs detection showed a much higher sensitivity and specificity with a much higher discriminative powers in the diagnosis of AMAN. When we analyzed the possible contribution of each analyte to the diagnosis of AMAN, only anti-GM1 AGA improved the sensitivity of the antibody testing. Several studies have reported an association between AMAN and IgG antibodies against GM1 and GD1a (16–18). In our study, GM1-IgG AGA was identified in five AMAN cases and GT1a-IgGAGA in three AMAN cases. The comparison of AMAN with non-AMAN patients with GBS showed that GM1-IgG AGA is strongly associate with AMAN. This illustrates that serum AGAs commonly accompanies the electrophysiological diagnosis of motor axonal dysfunctions, but not AIDP, the demyelinating variant which is the consequence of Schwann cell and myelin sheath injuries (19). In our study, AGAs were also detected in some patients with MFS. The same results were reported by Kusunoki et al. (20) who showed that IgG anti-GQ1b antibodies were positive in about 90% of the patients with MFS. Rinaldi et al. (21) reported a higher frequency of AGAs in acute inflammatory demyelinating polyneuropathy (AIDP) using the combinatorial glycoarray technique. However, a large study from Asia did not find excessive positivity of AGAs in AIDP (22, 23). In summary, IgG-AGAs has great potential to be a diagnostic protein biomarker for AMAN.

## Conclusion

Our research suggests that immunoblotting assay is convenient and feasible for simultaneous detection of the serum antibodies against multiple analytes. This method is suitable for the large-scale screening of patients with peripheral nervous system diseases. Despite the small size, the advantage of our study is that it is based on a well-established cohort of patient with a highly deterministic diagnosis. Future studies will attempt to confirm our observations in the lager cohorts.

## Data Availability Statement

The original contributions presented in the study are included in the article/supplementary material, further inquiries can be directed to the corresponding authors.

## Ethics Statement

The studies involving human participants were reviewed and approved by First Affiliated Hospital of Fujian Medical University. The patients/participants provided their written informed consent to participate in this study.

## Author Contributions

JZ, YZ, and GX formulated the study concept and acquired funding of the study. GX, ZZ, and NW designed the study. YZ, RL, YL, YF, NW, WZ, and YY collected and analyzed the data. JZ and YZ wrote the manuscript. All authors contributed to the article and approved the submitted version.

## Funding

This study was supported by grants from the National Natural Science Foundation of China (U2005201) and National Natural Science Foundation of China (82001287).

## Conflict of Interest

The authors declare that the research was conducted in the absence of any commercial or financial relationships that could be construed as a potential conflict of interest.

## Publisher's Note

All claims expressed in this article are solely those of the authors and do not necessarily represent those of their affiliated organizations, or those of the publisher, the editors and the reviewers. Any product that may be evaluated in this article, or claim that may be made by its manufacturer, is not guaranteed or endorsed by the publisher.
